# Genome sequence of *Roseovarius pacificus* strain IR27, a denitrifying bacterium from anoxic marine sediments with multiple methylamine oxidation pathways

**DOI:** 10.1128/mra.00884-25

**Published:** 2025-11-24

**Authors:** Isabel M. L. Rigutto, Thomas E. Noël, Theo A. van Alen, Geert Cremers, Mike S. M. Jetten

**Affiliations:** 1 Department of Microbiology, Radboud Institute for Biological and Environmental Sciences, Radboud University 6029 , Nijmegen, the Netherlands; California State University San Marcos, San Marcos, California, USA

**Keywords:** *Roseovarius*, genome, methylamine, nitrous oxide, marine, sediment

## Abstract

We report the draft genome sequence of *Roseovarius pacificus* strain IR27, isolated from anoxic marine sediments on monomethylamine and nitrous oxide. The assembled chromosome is 3,527,725 bp (62.56% GC), and the plasmid is 272,010 bp (59.18% GC). The genome contains denitrification as well as monomethylamine oxidation genes.

## ANNOUNCEMENT

*Roseovarius* is a genus within the family *Rhodobacteraceae*, whose members are found in diverse marine habitats (1, 2). We isolated strain IR27 from sediment (5–10 cm depth, September 2021) from the seasonally anoxic marine Lake Grevelingen (51.742°N, 3.849°E [3, 4]). Under anoxic conditions, sediment was mixed 1:10 with artificial seawater (ASW [3]) with 0.5 mM monomethylamine (MMA) as the sole carbon and nitrogen source and 2% nitrous oxide (N_2_O) as the electron acceptor. The bottles were incubated at ~22°C at 100 rpm, and substrates were resupplemented upon depletion. After several transfers into a modified ASW medium (t-ASW) containing 0.050 mM K_2_HPO_4_, 0.010 mM Na_2_S, 1 mL/L trace elements (DSMZ SL-10, modifications: 20 mg/L H_3_BO_3_, 170 mg/L CuCl_2_ · 2H_2_O, 67 mg/L SeO_2_, 240 mg/L CeCl_3_ · 7H_2_O, 50 mg/L Na_2_WO_4_ · 2H_2_O), and 1 mL/L vitamin solution (DSMZ 141, modifications: 0.2 mg/L vitamin B_12_), the enriched culture was spread on t-ASW agar (15 g/L) and 1.2 mM MMA. The plates were incubated at ~22°C in gas-tight containers with an argon headspace and 4.5% N_2_O. Strain IR27 was obtained by restreaking based on colony morphology, and its genome was sequenced to explore its metabolic capacity.

Genomic DNA was extracted from several colonies using the DNeasy PowerSoil Pro Kit (Qiagen, Venlo, the Netherlands), and sequenced using Nanopore long-read technology. DNA library construction was performed using the Native Barcoding Kit 24 V14 (SQK-NBD114-24; Oxford Nanopore Technologies, Oxford, UK) according to the manufacturer’s instructions but with AMPure XP beads added to the sample in a 0.4:1.0 vol ratio. The library was loaded on a Flow Cell (R10.4.1) and sequenced using the MinION Mk1C device. Basecalling was done with Dorado (model dna_r10.4.1_e8.2_400bps_sup@v5.0.0). In total, 482,977 reads were obtained, and their quality was analyzed using FastQC (v.0.11.9, https://www.bioinformatics.babraham.ac.uk/projects/fastqc/). The reads (N50: 7,887) were trimmed using BBduk (BBTools v.39.19; qtrim = rl, trimq = 20, minlen = 100) and assembled using Flye (v.2.9; --nano-raw, --meta [5]). The Flye output indicated that the resulting draft genome comprises a circular chromosomal contig (3,527,725 bp; 62.56% GC) with 734-fold coverage and a second smaller, circular contig (272,010 bp; 59.18% GC) with a coverage of 815. Quality assessment of the chromosome with CheckM (v.1.2.2 [6]) indicated that the assembly is 99.25% complete with 0.00% contamination. Taxonomic assignment using the Genome Taxonomy Database Toolkit (v.2.4.1 [7]) identified the isolate as belonging to *Roseovarius pacificus*, supported by an average nucleotide identity score of 95.93% when compared to the *R. pacificus* type strain genome (GCA_900142665.1) by fastANI (v.1.33 [8]). The genome was annotated using the NCBI Prokaryotic Genome Annotation Pipeline (6.10 [9]) and contained in total 3,772 predicted genes. The presence of plasmid replication genes (*repABC*) on the smaller contig confirms it as a plasmid, a feature previously observed in *R. pacificus* and other *Roseovarius* species (2, 10).

The genome contains gene clusters for the denitrification of nitrite to dinitrogen gas, including cytochrome cd1 nitrite reductase, nitric oxide reductase, and nitrous oxide reductase. The plasmid also harbors a copper-containing nitrite reductase. No nitrate reductase cluster was identified. Similar to other *Roseovarius* strains, strain IR27 can link denitrification to MMA oxidation. The genome contains three separate gene clusters for sarcosine oxidase (*soxABCD*), which is central to the N-methylglutamate pathway (11). It also encodes the glycine cleavage pathway and a comprehensive set of genes for folate-mediated one-carbon metabolism.

## Data Availability

This Whole Genome Shotgun project has been deposited at DDBJ/ENA/GenBank under accession number JBPZSF000000000. The version described in this paper is version JBPZSF010000000. The raw sequencing data are deposited under SRA accession number SRR34854828.
